# Adherence to a Mediterranean Diet is associated with physical and cognitive health: A cross-sectional analysis of community-dwelling older Australians

**DOI:** 10.3389/fpubh.2022.1017078

**Published:** 2022-11-16

**Authors:** Lisa Allcock, Evangeline Mantzioris, Anthony Villani

**Affiliations:** ^1^School of Health and Behavioral Sciences, University of the Sunshine Coast, Maroochydore, QLD, Australia; ^2^Clinical and Health Sciences and Alliance for Research in Exercise, Nutrition and Activity (ARENA), University of South Australia, Adelaide, SA, Australia

**Keywords:** Mediterranean diet, physical function, cognition, aging, iADLs

## Abstract

Poor cognitive function is associated with reduced functional independence, risk of institutionalization and reduced health-related quality of life. The ability to independently perform instrumental activities of daily living (iADLs) is compromised in patients with mild cognitive impairment (MCI) or dementia. Emerging evidence suggests that adherence to a Mediterranean diet (MedDiet), may play an important protective role against cognitive decline and dementia risk, whilst preserving functional status. This cross-sectional study aimed to explore the independent associations between MedDiet adherence, cognitive risk, and functional status in community-dwelling older adults living in Australia. MedDiet adherence was assessed using the Mediterranean Diet Adherence Screener (MEDAS); a modified Lawton's iADL scale was used for the assessment of functional status and risk of cognitive impairment was assessed using the AD8 dementia screening intervention. A total of *n* = 294 participants were included in the final analyses (70.4 ± 6.2 years; Females, *n* = 201; Males, *n* = 91; *n* = 2 unspecified). Adherence to a MedDiet was positively associated with functional ability (β = 0.172; CI: 0.022, 0.132; *P* = 0.006) independent of age, gender, Body Mass Index (BMI), smoking status, sleep duration, physical activity duration, diabetes status, and level of education. Furthermore, MedDiet adherence was inversely associated with cognitive risk (β = −0.134; CI: −0.198, −0.007; *P* = 0.035) independent of all covariates. However, our sensitivity analyses further showed that adherence to a MedDiet was not associated with cognitive risk in older adults free from cognitive impairment. We showed that adherence to a MedDiet is associated with healthy physical and cognitive aging. Nevertheless, exploration of these findings in larger cohorts, using longitudinal analyses and controlling for important confounders to ascertain the direction of the relationship is warranted.

## Introduction

Normal cognitive function is an essential component of healthy aging, affecting functional independence, risk of institutionalization and health-related quality of life (1, 2). Cognitive function is an umbrella term encompassing a number of distinct cognitive abilities related to processing speed, working memory, episodic memory, spatial ability, and learning (3). In general, levels of cognitive function increase normatively during childhood before peaking during adulthood, and eventually declining into old age (3). Distinct from cognitive impairment, dementia refers to a decline in mental ability that is severe enough to impact instrumental activities of daily living (4). Dementia is used to describe a collection of symptoms including loss of memory, intellect, rationality, social skills, and physical functioning (5). Interventions targeted at reducing dementia risk can be achieved through primary prevention, focusing on lowering risk factors for cognitively normal individuals or through secondary prevention aimed at high-risk individuals beginning to experience subjective cognitive decline (SCD) or mild cognitive impairment (MCI) (6). Risk factors for cognitive decline include age, cardiometabolic complications including diabetes, heart disease, hypertension, increased Body Mass Index (BMI) and central adiposity, smoking, sleep duration and sociodemographic factors such as sex, race/ethnicity, and education status (5, 7–9).

Mobility disability, physical frailty, and the inability to perform routine daily activities are important risk factors for dementia incidence or MCI (10–12). In the UK Biobank study, a large prospective cohort study with data available on a wide range of potential confounders, both slow gait speed and low grip strength (two important phenotypic characteristics of frailty) made the largest contributions to dementia incidence (12). Nevertheless, irrespective of how frailty is defined, the ability to independently perform basic activities of daily living (BADLs) (e.g., bathing, toileting, dressing, and feeding) and instrumental activities of daily living (iADLs) (e.g., handling finances, shopping, and food preparation) are compromised in patients with MCI or dementia risk (13–16). One proposed mechanism that potentially explains the relationship between physical frailty and cognitive decline with age is chronic inflammation (17–19). Aging is associated with immune dysregulation, characterized by an up-regulation of pro-inflammatory cytokines, including interleukin 1 (IL-1), IL-6, IL-8, tumor necrosis factor alpha (TNF-α), and C-reactive protein (CRP), a phenomenon termed inflammageing (20, 21). High levels of these pro-inflammatory markers in the blood and other tissues are predictive of physical frailty and cognitive decline (18). As such, lifestyle strategies (e.g., physical activity, nutrition and behavioral) targeted at attenuating inflammation and reducing the risks associated with functional and cognitive decline are of upmost importance to prevent or delay the onset of such conditions.

Epidemiological evidence suggests that adherence to plant-based, anti-inflammatory dietary patterns may play an important protective role against cognitive decline and dementia risk (22, 23), whilst preserving functional status (24, 25). More specifically, adherence to a Mediterranean diet (MedDiet), which is often heralded as an anti-inflammatory dietary pattern, has been positively associated with healthy cognitive functioning (7, 26–29) and attenuates frailty risk in older adults (30, 31). The traditional MedDiet is consistent with a dietary pattern and time-honored eating behaviors by populations living in the olive-tree growing areas of the Mediterranean basin before the mid-1960's (26, 32). Although challenging to define given that a single MedDiet does not exist (33, 34), the MedDiet is often described in the literature as a plant-based dietary pattern, consistent with a high intake of vegetables, fruits, nuts, legumes, wholegrains cereals, and daily use of extra-virgin olive oil incorporated into all meals; moderate consumption of fish, shellfish, fermented dairy products (cheese and yogurt), and wine (typically during meals); and a low or infrequent consumption of meat and processed meat products, processed cereals, sweets, vegetable oils, and butter (7, 26, 32). In recent years, adherence to the MedDiet has been widely investigated and promoted as one of the ‘healthiest' dietary patterns with respect to reductions in chronic disease risk and healthy aging (35, 36) including studies conducted in non-Mediterranean countries, such as Australia (37–40). However, whether such findings are consistent with cognitive function and activities of daily living (ADLs) in non-institutionalized older adults from non-Mediterranean countries warrants exploration.

Therefore, this cross-sectional study investigated the independent associations between adherence to a MedDiet, risk of cognitive impairment and functional status in community-dwelling older adults from Australia. As secondary analyses, we also explored the independent associations between individual MedDiet food components [as defined by the Mediterranean Diet Adherence Screener (MEDAS)], cognitive impairment and functional status in the same cohort of older adults.

## Methods

### Participants and recruitment

A cross-sectional study was undertaken amongst community-dwelling older adults ≥60 years. Older Australians who were permanent residents of Australia, free from dementia or cognitive decline and could independently complete an anonymous online survey in English were invited to participate. Participants were recruited via social media platforms including Facebook, Twitter, Instagram, and LinkedIn, and networking with the Local Government Councils from February 2022 to May 2022 requesting voluntary participation. The investigators disseminated the survey link using the aforementioned social media platforms weekly across these dates. Qualtrics™ survey software was used to construct and distribute the survey. A link to the survey was disseminated via social media platforms where the study protocol and potential risks were clearly outlined to all interested participants. Exclusion criteria included those <60 years of age, those with dementia or cognitive decline and/or unable to complete the online survey in English and/or did not permanently reside in Australia. This study was conducted according to the guidelines described in the Declaration of Helsinki and was approved by the Research Ethics Committees at the University of the Sunshine Coast (S221680) and the University of South Australia (204450). Participants acknowledged an informed consent statement prior to participation in the study.

### Data collection

A 75-item self-administered questionnaire was used to assess the relationship between adherence to a MedDiet, risk of cognitive impairment and ADLs. The questionnaire was divided into six separate sections and included previously validated tools including the Lawton's iADLs scale (41, 42), AD8 dementia screening intervention (43–45), Depression Anxiety Scale (DASS-21) (46) and the MEDAS (47, 48). An additional four questions pertaining to eating behaviors and socialization characteristics related a traditional MedDiet lifestyle were also adapted from the MediCul tool (49). The questionnaire also consisted of open and closed-ended questions related to participant demographic characteristics, which included self-reported height and weight, presence of disease, medications, physical activity status, sleep duration and smoking status. The questionnaire was designed to be completed in ~30 min. There were no time restrictions applied to complete the questionnaire and participants were not required to answer all questions before proceeding to subsequent questions. The link to the survey was recognizable once only to the server it was sent thus preventing duplication when responding to the survey. The investigators also screened all of the participant responses (IP address and postcode viewed) to ensure all responses were consistent with the eligibility criteria. For the purpose of the present study, we will report on data from the MEDAS, Lawton's iADLs and the AD8 dementia screening intervention.

### Instrumental activities of daily living

The modified Lawton iADL scale was used to assess functional ability (41). This previously validated tool comprised of eight questions assessing instrumental activities including: *ability to use the telephone, shopping, food preparation, housekeeping, laundry, mode of transportation, responsibility for own medications* and *ability to handle finances* (41, 42). For each question, participants were asked to select the option that closely resembles their highest level of function. Each question was rated a dichotomized value (0 for less able and 1 for more able). The aforementioned eight items were summed to form a score ranging from 0 to 8, with 8 representing no disability in iADLs.

### Cognitive function

Cognitive function was assessed using the self-administered AD8 dementia screening intervention which is a validated instrument to help discriminate between signs of normal cognition and mild dementia (43). The tool is comprised of eight items which assess cognitive abilities related to *memory, orientation, judgment*, and *executive function* (44). For each of the items, participants were asked to rate if they had noticed any changes in the last few years. Each question was rated in accordance with three possible categories: yes, a change; no change; do not know. A score of one for each ‘yes, a change' and zero for each ‘no change' or ‘do not know' were applied. As such, higher scores are suggestive of cognitive decline (44, 45). Specifically, a score ≥2 is suggestive of cognitive impairment.

### Mediterranean diet adherence

Adherence to a MedDiet was assessed using the validated 14-item MEDAS (48), which was developed and used in the Prevención con Dieta Mediterránea (PREDIMED) study (47). The MEDAS includes 12 questions which assess the main dietary elements of a traditional MedDiet, and two questions related to food intake behaviors that are consistent with a MedDiet (48). Each of the 14 questions is scored dichotomously as 0 or 1, generating a maximum score of 14 where higher scores are reflective of greater adherence to a MedDiet (48). Specifically, for food intake behaviors consistent with a MedDiet pattern, one point was awarded if participants identified using olive oil as their main source of dietary fat; one point was also awarded for preferentially consuming white meat over red meat. For the dietary elements consistent with a traditional MedDiet, one point was awarded if participants met the serve size and frequency of consumption criteria for the following food components:

- Four or more tablespoons (1 tablespoon = ~15 g) of olive oil per day (including use in frying, salads, meals consumed away from the home etc);
- Two or more servings of vegetables per day (1 x serve equates to 2 x cups vegetables);
- Three or more pieces of fruit per day (inclusive of whole, tinned or dried fruit but excludes fruit juice);
- Fewer than one serving of red meat or sausages per day (1 x serve equates to 100–150 grams);
- Fewer than one serving of butter, margarine or cream per day (1 x serve equates to 10 grams);
- Fewer than one cup (250 ml) of sugar-sweetened beverages per day;
- Seven or more servings of red wine per week (1 x serve equates to 100 ml);
- Three or more servings of legumes per week (1 x serve equates to 1 x cup);
- Three or more servings of fish or seafood per week (1 x serve equates to 100–150 grams)
- Fewer than three commercial sweets or pastries (not homemade) such as cakes, cookies and biscuits per week;
- Three or more servings of nuts (including peanuts) per week (1 x serve equates to 30 grams or ~1 x handful);
- Two or more servings per week of a dish (e.g., pasta / rice / vegetables) made with a traditional sauce including tomatoes, garlic, onion, or leeks sautéed in olive oil.

### Statistical analysis

All continuous variables were presented as means ± standard deviation (SD) with categorical data presented as frequencies and percentages. The Kolmogorov–Smirnov statistic was used to assess normality of data prior to all tests, and multiple regression diagnostics were performed to ensure assumptions of multicollinearity and homoscedasticity were not violated. Independent samples *t*-tests were used to explore differences in demographic characteristics between genders. Pearson's correlation coefficients were used to identify the independent associations between adherence to a MedDiet, cognitive risk and iADLs. Univariable and multivariable linear regression analysis (and 95% CI) were also used to investigate the independent association between adherence to a MedDiet, cognitive risk and iADLs using one unadjusted and six adjusted predictor models. We also applied univariable and multivariable regression analysis on individual dietary elements consistent with a traditional MedDiet to explore their independent relationships on cognitive risk and iADLs. Covariates included in our predictor models included age, gender, BMI, smoking status, sleep duration, physical activity status, presence of diabetes and level of education. Standardized beta-coefficients were used in the univariable and multivariable linear regressions with z-scores for all outcome variables calculated before running each of the regression models to ensure comparisons across each of the outcomes. Therefore, the beta-coefficients are interpreted as the change in the predicted value for each of the outcomes based on a SD increase in the MEDAS. We also conducted additional sensitivity analyses to assess the robustness of our results. Specifically, we replicated our univariable and multivariable linear regression analysis on participants free from cognitive impairment (e.g., AD8 score <2) to examine the independent association between adherence to a MedDiet (and individual dietary elements of the MedDiet), cognitive risk and iADLs. Analyses were performed using Statistical Package for the Social Sciences (SPSS) for Windows 26.0 software (IDM Corp., Armonk, NY, USA), with statistical significance set a *P* < 0.05.

## Results

A total of *n* = 303 community-dwelling older adults undertook the online questionnaire (Females, *n* = 205; Males, *n* = 96; *n* = 2 unspecified). A total of *n* = 294 participants (Females, *n* = 201; Males, *n* = 91; *n* = 2 unspecified) completed all components of the online questionnaire which was used in the final analysis. Demographic characteristics of the participants are presented in Table 1. The majority of participants mobilized independently without the need for mobility aids and did not receive domiciliary care services within the home. According to iADL and AD8 scores, the total sample displayed a high functional ability and independence (7.68 ± 0.93; range: 0–8) and normal cognitive functioning (1.07 ± 1.580; range: 0–8). However, a total of *n* = 79 participants (~27%) were at risk of cognitive impairment, as defined by the AD8 score (e.g., AD8 score ≥2). Independent samples *t*-test showed significant gender differences in MedDiet adherence scores (Females: 5.85 ± 1.90; Males: 4.91 ± 2.22; *P* = <0.001) and functional ability (Females: 7.78 ± 0.80; Males: 7.47 ± 1.12; *P* = 0.009). No significant differences in cognitive risk between genders was observed.

**Table 1:** Participant demographic characteristics by gender^*^.

**Characteristics**	**Total**	**Male**	**Female**
Age (years)	70.4 ± 6.2	72 ± 6.9	69.67 ± 5.8
Gender *n*, %		96 (31.7)	205 (67.7)
BMI (kg/m2)	28.8 ± 7.2	29.1 ± 8.7	28.8 ± 6.5
Education status ***n*****, %**			
No schooling completed	1 (0.3)	1 (0.3)	0 (0.0)
Junior or primary school	6 (2.0)	5 (5.2)	1 (0.5)
Secondary school	58 (19.1)	11 (11.5)	45 (22.0)
Trade/technical/vocational training	60 (19.8)	24 (25.0)	36 (17.6)
Diploma	56 (18.5)	18 (18.8)	38 (18.5)
Advanced diploma/associate degree	22 (7.3)	4 (4.2)	18 (8.8)
Bachelor's degree	54 (17.8)	18 (18.8)	36 (17.6)
Postgraduate degree or doctorate	45 (14.9)	15 (15.6)	30 (14.6)
**Level of mobility** ***n*****, %**			
Independent without the use of any aids	279 (92.1)	87 (90.6)	190 (92.7)
Mostly dependent on a walking stick	15 (5.0)	8 (8.3)	7 (3.4)
Dependent on a four-wheeled walker	4 (1.4)	0 (0.0)	4 (2.0)
Dependent on a scooter	3 (1.0)	1 (1.0)	2 (1.0)
**Services** ***n*****, %**			
No services, fully independent	268 (88.4)	83 (86.5)	183 (89.3)
Domiciliary care	17 (5.6)	4 (4.2)	13 (6.3)
Other (including DVA, local council, TCP residential/community, MOWs)	18 (5.9)	9 (9.4)	9 (4.4)
**Diabetes status** ***n*****, %**			
No diabetes present	260 (85.8)	78 (81.3)	180 (87.8)
Type 2 diabetes mellitus	30 (9.9)	16 (16.7)	14 (6.8)
Type 1 diabetes mellitus	2 (0.7)	0 (0.0)	2 (1.0)
**Smoking status** ***n*****, %**			
Non-smoker	163 (53.8)	47 (48.0)	114 (55.6)
Former smoker	125 (41.3)	45 (45.9)	80 (39.0)
Current smoker	12 (4.0)	4 (4.1)	8 (3.9)
Physical activity duration (min/day)	94.8 ± 81.1	85.6 ± 74.7	98.5 ± 83.9
Sedentary activity duration (min/day)	381.1 ± 171.7	390.3 ± 170.8	375.1 ± 172.0
Sleep duration (min/night)	405.5 ± 73.3	414.6 ± 66.3	402 ± 78.2
**Sleep quality** ***n*****, %**			
Very bad	7 (2.3)	2 (2.1)	5 (2.4)
Fairly bad	76 (25.1)	15 (15.6)	60 (29.3)
Fairly good	172 (56.8)	60 (62.6)	111 (54.1)
Very good	48 (15.8)	19 (19.8)	29 (14.1)
Functional ability (iADL)	7.68 ± 0.9	7.47 ± 1.1	7.78 ± 0.8
Cognitive risk (AD8)	1.07 ± 1.6	1.1 ± 1.5	1.1 ± 1.6
MedDiet adherence (MEDAS)	5.57 ± 2.1	4.91 ± 2.2	5.85 ± 1.9

* Two participants did not disclose gender but are included in the total column.

BMI, Body Mass Index; DVA, Department of Veterans Affairs; TCP, Transitional Care Program; MOWS, Meals on Wheels; iADL, instrumental Activities of Daily Living; AD8, AD8 dementia screening intervention; MedDiet, Mediterranean diet; MEDAS, Mediterranean diet adherence screener.

Overall, the entire sample reported moderate adherence to a MedDiet (5.57 ± 2.059; range: 1–11). Table 2 presents descriptive data derived from the MEDAS on the proportion of participants achieving normative criteria for each individual MEDAS question. Use of olive oil (*n* = 211, 69.6%), achieving recommended serves of vegetables (*n* = 211, 69.6%), low reported frequency of sugar-sweetened beverages (*n* = 224, 73.9%), and low reported frequency of commercial sweets and pastries (*n* = 222; 73.3%) were the only dietary elements included in the MEDAS whereby at least half of all participants achieved recommended serve size criteria.

**Table 2:** Proportion of participants achieving normative criteria for each individual MEDAS question.

**MedDiet food components**	**Achieved *n*, (%)**
Olive oil for cooking	211 (69.6)
Olive oil consumed	4 (1.3)
Vegetable serves	211 (69.6)
Fruit serves	77 (25.4)
Red and processed meat	82 (27.1)
Butter, margarine, cream	69 (22.8)
Sugar-sweetened beverages	224 (73.9)
Wine	19 (6.3)
Legumes	59 (19.5)
Fish	41 (13.5)
Pastry	222 (73.3)
Nuts	118 (38.9)
Preference for white meats	200 (66.0)
Tomato-based dishes	136 (44.9)

MedDiet, Mediterranean diet; MEDAS, Mediterranean diet adherence screener.

Pearson's correlation coefficients showed a weak, yet positive association between adherence to a MedDiet and functional ability (*r* = 0.228; *P* = <0.001), and an inverse association between MedDiet adherence and cognitive risk (*r* = 0.148; *P* = 0.011). Table 3 displays standardized beta coefficients (and 95% confidence intervals) from univariable and multivariable linear regression analysis for independent associations between MedDiet adherence, functional ability, and cognitive risk. Adherence to a MedDiet was positively associated with iADLs (β = 0.172; CI: 0.022, 0.132; *P* = 0.006) independent of age, gender, BMI, smoking status, sleep duration, physical activity duration, diabetes status, and education status. Furthermore, MedDiet adherence was inversely associated with cognitive risk (β = −0.134; CI: −0.198, −0.007; *P* = 0.035) independent of all covariates used in the fully adjusted model.

**Table 3:** Univariable and multivariable linear regression coefficients (and 95% CI) expressing independent associations between adherence to a Mediterranean diet, functional ability (iADL), and cognitive risk (AD8).

**Model**	**Cognitive risk (AD8)**		**Functional ability (iADL)**	
	**Beta**	***P***	**Beta**	***P***
1^a^	−0.148 (−0.203, −0.026)	0.011	0.228 (0.051, 0.150)	<0.001
2^b^	−0.152 (−0.209, −0.028)	0.011	0.209 (0.042, 0.142)	<0.001
3^c^	−0.161 (−0.213, −0.031)	0.009	0.203 (0.038, 0.140)	<0.001
4^d^	−0.138 (−0.199, −0.013)	0.026	0.188 (0.030, 0.136)	0.002
5^e^	−0.134 (−0.195, −0.010)	0.031	0.183 (0.028, 0.134)	0.003
6^f^	−0.134 (−0.198, −0.007)	0.035	0.172 (0.022, 0.132)	0.006
7^g^	−0.134 (−0.198, −0.007)	0.035	0.172 (0.022, 0.132)	0.006

iADL, instrumental Activities of Daily Living; AD8, AD8 dementia screening intervention; Beta, Standard beta coefficient; Standardized beta coefficient represents the change in a SD-unit increase in the Mediterranean Diet Adherence Screener score per change in outcome measure.

a Non-adjusted model.

b Adjusted for age and gender.

c Adjusted for age, gender, and BMI.

d Adjusted for age, gender, BMI, smoking status, and average sleep duration/night.

e Adjusted for age, gender, BMI, smoking status, average sleep duration/night and average physical activity duration/day.

f Adjusted for age, gender, BMI, smoking status, average sleep duration/night, average physical activity duration/day, and diabetes status.

g Adjusted for age, gender, BMI, smoking status, average sleep duration/night, average physical activity duration/day, diabetes status and education status.

When we assessed individual dietary elements included in the MEDAS, we showed that nut intake and consumption of sugar-sweetened beverages (<250 ml per day) were both inversely associated with cognitive risk, independent of all covariates in the fully adjusted model (nuts: β = −0.131; CI: −0.828, −0.024; *P* = 0.038; sugar-sweetened beverages: β = −0.170; CI: −1.075,−0.194; *P* = 0.005). Furthermore, vegetable intake and consumption of sugar-sweetened beverages (<250 ml per day) were both positively associated with iADLs in our fully adjusted model (vegetables: β = 0.173; CI: 0.116, 0.589; *P* = 0.004; sugar-sweetened beverages: β = 0.137; CI: 0.041, 0.554; *P* = 0.023). No other significant findings for any other individual dietary element included in the MEDAS were observed.

We also performed independent univariable and multivariable regression analyses in participants free from cognitive impairment. Our sensitivity analyses showed that adherence to a MedDiet remained positively associated with iADLs, independent of age, gender and BMI (β = 0.156; CI: 0.005, 0.090; *P* = 0.03). However, this association was no longer significant after controlling for smoking status, sleep duration, physical activity duration, diabetes status, and level of education. Our sensitivity analyses further revealed that adherence to a MedDiet was not associated with cognitive risk in participants with an AD8 score <2 (β = −0.043; CI: −0.039, 0.020; *P* = 0.532). When assessing individual dietary elements from the MEDAS, after removing participants at risk of cognitive impairment, no significant relationship between nut consumption (β = −0.111; CI: −0.223, 0.20; *P* = 0.101) and sugar-sweetened beverages (β = −0.018; CI: −0.165, 0.126; *P* = 0.791) were observed. However, consumption of <250 ml (1 x cup) of sugar-sweetened beverages per day and vegetable consumption both remained positively associated with iADLs using our fully adjusted model in our sensitivity analyses in participants free from cognitive decline (sugar-sweetened beverages: β = 0.221; CI: 0.135, 0.583; *P* = 0.002; vegetables: β = 0.210; CI: 0.101, 0.500; *P* = 0.003).

## Discussion

The primary aim of this study was to explore the independent associations between adherence to a MedDiet, risk of cognitive impairment and functional status in Australian community-dwelling older adults. We showed that adherence to a MedDiet was positively associated with functional ability and inversely associated with cognitive risk, independent of age, gender, BMI, smoking status, sleep duration, physical activity duration, diabetes status, and level of education. However, our sensitivity analyses further showed that adherence to a MedDiet was not associated with cognitive risk in older adults free from cognitive impairment (e.g., AD8 score <2). We also observed that low consumption of sugar-sweetened beverages (<250 ml per day) and increased vegetable intake (≥2 cups per day), as defined by the MEDAS, were both positively associated with iADLs, independent of all covariates.

Our findings are indeed consistent with previous literature suggesting that adherence to a MedDiet is positively associated with self-reported ADLs (50, 51). Unlike our findings, in a community-based, multiethnic longitudinal study of older adults (*n* = 1,696) aged ≥65 years from the United States, Guo et al. (52) reported that higher levels of adherence to a MedDiet was inversely associated with disability in accordance with BADL but not for iADLs. However, this inverse association was not observed across all multiethnic groups and no longer significant when adjusted for smoking status and co-morbidities such as hypertension, diabetes, and heart disease. In the present study, the level of significance was maintained across a broad range of covariates including smoking status and self-reported diabetes. Importantly however, we did not adjust for ethnicity or other chronic diseases known to influence functional status and risk of disability, such as cardiovascular disease (53). Furthermore, unlike the aforementioned studies, we used different instruments to assess MedDiet adherence and ADLs. As such, any observed differences in associations between MedDiet adherence and ADLs can at least be partially explained by the marked heterogeneity (e.g., study population, study design, length of follow-up, assessment tools, residual confounders etc) across different studies. Nevertheless, there is indeed overwhelming evidence to support that adherence to a MedDiet is inversely associated with incident frailty risk (30, 31) and characteristics of the physical frailty phenotype, in particular gait speed (54–57).

We further showed that MedDiet adherence was inversely associated with cognitive risk in community-dwelling older adults. These findings are consistent with recent meta-analytical data supporting that high adherence to a MedDiet is cross-sectionally and longitudinally associated with overall cognitive function, MMSE scores and memory in older adults free from dementia (58). These findings have also been demonstrated in non-Mediterranean countries, including the United States and Australia (59, 60). In a longitudinal analysis of community-dwelling adults living in Central New York (MSLS study), Wade et al. (59) reported longitudinal associations between MedDiet adherence and global cognitive and executive functioning in older adults ≥70 years. Similarly, in a cross-sectional analysis of the Sydney Memory and Aging Study (MAS), Chen et al. (60) reported that adherence to a MedDiet was positively associated with visuospatial cognition. Nevertheless, these findings are not all consistent. A recent systematic review by Limongi et al. (61) suggested that whilst adherence to a MedDiet may indeed be associated with improved cognitive health, its relationship with other specific cognitive domains (e.g., memory, language, executive function) remains unclear, likely due to the heterogeneity in the modalities used to assess cognitive outcomes, thus making the interpretation of the findings challenging. Importantly, methodological differences in how the MedDiet is operationalized is also likely to contribute to inconsistent findings. As such, many of the aforementioned studies assessed adherence to a MedDiet using (or an adaptation of) the Mediterranean Diet Score developed by Trichopoulou et al. (62) which is dependent on the habitual dietary characteristics of the studied population and may not reflect true adherence to a MedDiet, particularly in non-Mediterranean countries (63). In the present study, we assessed adherence to the MedDiet using the MEDAS, which is based on normative criteria and reflective of a Mediterranean-style diet.

Given the heterogeneity and potential limitations associated with MedDiet adherence tools, examining individual dietary constituents of the dietary pattern may assist in standardizing research methodologies and help quantify intake of specific food groups to ensure maximum health-related benefits. In the present study, we showed that nut consumption (as defined by the MEDAS) was inversely associated with cognitive risk, independent of age, gender, BMI, smoking status, sleep duration, physical activity duration, diabetes status, and level of education. These results are consistent with the MAS study (60) which showed significant positive associations between nut consumption and better global cognition and higher scores in multiple cognitive domains, including processing speed, language, visuospatial and executive functioning. Using cross-sectional data from the National Health and Nutrition Examination Surveys, Tan et al. (64) reported that moderate nut intake (15–30 grams per day) was associated with better cognitive performance in older adults with non-alcoholic fatty liver disease. However, a comprehensive systematic review found inconsistent evidence related to regular nut consumption and cognitive health in adults across a range of different ages (65). Although marked heterogeneity across individual studies was reported, more homogeneous findings were observed in studies examining the association between walnut consumption and cognitive performance, particularly in older adults. This is also supported from the results of the PREDIMED study which showed that a MedDiet supplemented mixed nuts (almonds, walnuts, and hazelnuts) or EVOO improved global cognitive performance after 6.5 years of the nutritional intervention when compared against a low-fat control group (47). Nevertheless, not all clinical trials have identified significant differences in cognitive performance in older adults after dietary interventions supplemented with nuts (66, 67). Of note, in our sensitivity analyses, the inverse relationship between nut consumption and cognitive risk observed in the present study was no longer significant in participants free from cognitive decline. Irrespective, nuts are nutrient dense, providing rich amounts of unsaturated fatty acids, fiber and antioxidants which contribute to a healthy dietary pattern, such as the MedDiet (68–70). As such, the underlying mechanisms attributable to nut consumption and better cognition are likely to be multifaceted, including improvements in endothelial function and enhanced cerebral blood flow, regulation of brain glucose concentrations and enhanced intestinal microbiota composition (71–73).

Further, we also observed an inverse relationship between sugar-sweetened beverage consumption and cognitive risk. In a subsample (*n* = 806) of the Seguimiento Universidad de Navarra (SUN) cohort, Munoz-Garcia et al. (74) also reported that the consumption of more than 1 sugar-sweetened beverage per month, compared to seldom consumption, was significantly associated with a decline in cognitive function after 6 years. However, unlike our study, the aforementioned study was a longitudinal analysis of sugar-sweetened beverage consumption from the previous year using a validated 136-item semi-quantitative food-frequency questionnaire. Moreover, the investigators also used an alternative instrument to assess cognitive risk [modified Telephone Interview of Cognitive Status (STICS-m)] and different frequency of consumption criteria to define sugar-sweetened beverage consumption, making comparisons of the study findings difficult. Our sensitivity analyses also showed that this relationship was no longer significant in participants free from cognitive decline in our study sample. However, we also observed that low consumption of sugar-sweetened beverages and increased vegetable intake (≥2 serves per day in accordance with the MEDAS) were both positively associated with iADLs. This finding was also consistent in our sensitivity analyses. These observations are also consistent with previous longitudinal (75) and meta-analytic analyses (76) related to sugar-sweetened beverage and vegetable consumption on the risks associated with physical frailty. Specifically, in older women (*n* = 71,935) participating in the Nurses' Health Study, Struijk et al. (75) showed that habitual consumption of ≥1 serving per day of sugar-sweetened beverages was associated with a higher risk of frailty during a 22-year follow-up. Moreover, a recent systematic review and meta-analysis of prospective cohort studies reported that greater vegetable consumption was associated with lower risk of incident frailty among community-dwelling older adults (76).

However, the paradigm of assessing dietary patterns as opposed to individual nutrients or single foods as a determinant of chronic disease risk has been recognized for some time (77). With respect to the MedDiet, there are several nutritive mechanisms associated with the beneficial effects of an anti-inflammatory dietary pattern on cognition and functional status (78–82). In particular, anti-inflammatory dietary patterns have been shown to reduce central adiposity, blood pressure and platelet aggregation, all of which are protective against atherosclerosis and improve vascular blood flow, leading to reduced cardiovascular risk and improved brain functioning (78, 80). In addition, oxidative stress and low-grade chronic inflammation are also observed with age-related chronic diseases, including cognitive decline and physical frailty (83–85). As such, the high antioxidant capacity of the MedDiet has a key role in modulating signaling pathways involved in the up regulation of pro-inflammatory mediators and reactive oxygen species (86), and therefore may play an important role in preserving physical and cognitive status with age. Lastly, the MedDiet is also rich in bioactive properties, including omega-3 fatty acids (87), which may have direct effects on gut microbiota, which has been shown to influence neurocognitive health (73, 78).

There are several limitations that must be considered when interpreting the results of this study. Firstly, the cross-sectional study design prevents causality from being determined. Additionally, our results may be overstated given that our sample were relatively healthy, independent, and free from mobility disability and cognitive impairment (as per iADL and AD8 scores). Nevertheless, our sample was not entirely homogenous given that one-quarter of the study sample were at risk of cognitive impairment, as defined by AD8 scores. Importantly, when we removed participants at risk of cognitive impairment in our sensitivity analyses, no significant relationship between MedDiet adherence (or individual MedDiet components) and cognitive risk was observed. This is indeed an interesting finding given that it could be hypothesized that nutrients and dietary patterns alike are more likely to exert a greater influence on cognition before the onset of cognitive decline or neurodegenerative diseases. However, this was not observed in the present study; nevertheless, by and large our participants were independent and relatively healthy and therefore may have a narrower range of normal cognitive function. Perhaps most pertinently, the limited scale which defines ‘normal cognition' (e.g., AD8 score <2) in the AD8 dementia screening tool that was used in the present study is likely to reduce the magnitude of the association in our sensitivity analyses. Further to this, the lack of association between adherence to a MedDiet and cognitive risk in participants free from cognitive decline may also be due to modest adherence scores, and in particular a lack of participants with high adherence, thus limiting the generalizability of our study findings. Moreover, it is unknown whether these findings are generalizable to more vulnerable populations at greater risk of functional and cognitive decline, such as institutionalized older adults. Given the nature of our study cohort and method of recruitment (e.g., participation in an online survey), the potential for selection bias is an important consideration in the interpretation of these study findings. In addition, although our multiple regression analyses were adjusted for important confounders, residual confounding cannot be ruled out. In particular, we did not consider nor capture all aspects of dietary intake. For example, western dietary patterns (higher in free sugars and saturated fat) have been found to be associated with risk of physical frailty and cognitive impairment, respectively (88–91). Further to this, we did not adjust for other important covariates associated with cognitive status and physical frailty such as total energy intake. There is also a possibility that our multiple regression analyses were over-adjusted, leading to selection bias and thus reducing the precision of the effect estimates (e.g., influence of MedDiet adherence) (92). Another important limitation was that dietary intake data and the iADL and AD8 instruments were self-reported thus increasing the potential for recall or social desirability bias. In addition, both the Lawton's iADL and AD8 dementia screening intervention instruments used in the present study are screening tools and are not typically used to assess mobility disability and cognitive impairment, respectively. Nevertheless, the AD8 has previously been validated against performance based cognitive screening assessments such as the MMSE and the Montreal Cognitive Assessment (MoCA) which are both commonly used as cognitive assessment tools (43). Moreover, the Lawton iADL scale has been widely used and is validated to assess functional status in older adults with or at risk of dementia (42).

In conclusion, these cross-sectional analyses demonstrated that adherence to a MedDiet was positively associated with functional status (iADL) and inversely associated with risk of cognitive decline in community-dwelling older adults. However, when participants at risk of cognitive impairment were removed in our sensitivity analysis, no significant relationship between MedDiet adherence and cognitive risk was observed. We also observed that certain dietary constituents of the MedDiet, namely a low consumption of sugar-sweetened beverages and increased vegetable intake, were independently associated with iADLs. As such, our results contribute to the growing body of evidence in support of the MedDiet for healthy physical and cognitive aging. Nevertheless, exploration of these findings in vulnerable populations, with a wider range of adherence scores, particularly higher adherence, using longitudinal analyses and controlling for important confounders in order to ascertain the direction of the relationship is warranted.

## Data availability statement

The data that support the findings of this study will be made available from the corresponding author upon reasonable request.

## Ethics statement

The studies involving human participants were reviewed and approved by University of the Sunshine Coast (S221680) and the University of South Australia (204450). The patients/participants provided their written informed consent to participate in this study.

## Author contributions

LA and AV were responsible for the study conception and design. All authors were responsible for data collection, statistical analysis, interpretation of the findings, and drafting the final manuscript.

## Funding

This work was supported by funding from the School of Health and Behavioral Sciences at the University of the Sunshine Coast. No external funding was awarded for this project.

## Conflict of interest

The authors declare that the research was conducted in the absence of any commercial or financial relationships that could be construed as a potential conflict of interest.

## Publisher's note

All claims expressed in this article are solely those of the authors and do not necessarily represent those of their affiliated organizations, or those of the publisher, the editors and the reviewers. Any product that may be evaluated in this article, or claim that may be made by its manufacturer, is not guaranteed or endorsed by the publisher.
